# A Novel Homozygous MYH2 Variant Causing Early-Onset External Ophthalmoplegia and Proximal Myopathy in a Consanguineous Family

**DOI:** 10.7759/cureus.111054

**Published:** 2026-06-17

**Authors:** Mustafa A Hammad, Wasef Alhroub, Ahmad Isaid

**Affiliations:** 1 Neurology, Hammad Neurologic Center, Ramallah, PSE; 2 General Medicine, Hammad Neurologic Center, Ramallah, PSE; 3 General Medicine, Hammad Neurologic Center, American Medical Institute, Ramallah, PSE

**Keywords:** congenital myopathy, consanguinity, external ophthalmoplegia, myh2, proximal muscle weakness

## Abstract

Mutations in the MYH2 gene, which encodes skeletal muscle myosin heavy chain IIa, are a rare cause of congenital myopathies characterized by proximal muscle weakness and ophthalmoplegia. MYH2-related disease may be inherited in either an autosomal dominant or autosomal recessive manner. Autosomal recessive MYH2-related myopathy is uncommon and may be difficult to recognize because of overlapping clinical features with neuromuscular junction disorders. We report a six-year-old boy born to consanguineous parents who presented with congenital bilateral ptosis, external ophthalmoplegia, and progressive non-fluctuating proximal muscle weakness. Neurological examination showed marked limitation of eye movements, proximal limb weakness, preserved reflexes, and normal cognitive development. Brain MRI and metabolic investigations were normal. Whole exome sequencing identified a novel homozygous MYH2 variant, c.1124A>C (p.Gln375Pro), while both parents were heterozygous carriers, consistent with autosomal recessive inheritance. The variant affects a highly conserved residue within the myosin motor domain and is absent from population databases. The clinical phenotype was consistent with previously reported cases of recessive MYH2-related myopathy. This case expands the mutational spectrum of MYH2-associated disease and highlights the importance of genetic evaluation in children presenting with congenital ophthalmoplegia and proximal muscle weakness.

## Introduction

Mutations in the MYH2 gene are a rare cause of congenital and early-onset myopathies, representing a spectrum of disease with both autosomal recessive and autosomal dominant inheritance patterns. These disorders are characterized by external ophthalmoplegia and predominantly proximal limb weakness. MYH2 encodes the myosin heavy chain IIa (MyHC-IIa), a protein highly expressed in fast type 2A skeletal muscle fibers that is essential for normal muscle contraction and structural integrity. Pathogenic variants in MYH2 give rise to distinct autosomal recessive and autosomal dominant phenotypes, which differ in age of onset, severity, and histopathological features [1].

Autosomal recessive MYH2 myopathy typically presents in early childhood with bilateral ptosis, external ophthalmoplegia, and non-progressive or slowly progressive limb weakness, with muscle biopsy often demonstrating reduced or absent type 2A fibers and decreased MyHC-IIa expression [1]. In contrast, dominant MYH2 variants generally present later and show greater phenotypic variability, with muscle biopsies occasionally demonstrating rimmed vacuoles; however, ophthalmoplegia remains a frequent clinical feature [2].

Here, we report a six-year-old boy with congenital bilateral ptosis, external ophthalmoplegia, and proximal muscle weakness in the setting of parental consanguinity. Genetic testing identified a novel homozygous MYH2 variant (c.1124A>C; p.Gln375Pro), classified as a variant of uncertain significance. This case highlights the phenotypic and genotypic spectrum of MYH2-related myopathies, particularly the autosomal recessive form, and underscores the importance of correlating genotype with clinical phenotype.

## Case presentation

The patient is a six-year-old boy, born via Cesarean section 10 days preterm to consanguineous parents (first cousins). His developmental milestones were largely within normal limits; he achieved independent ambulation at 15 months and demonstrated early language acquisition. His clinical history is significant for bilateral ptosis present since birth, with a history of dysphagia and seasonal cough. The dysphagia was mild and intermittent, primarily affecting solid foods. No episodes of aspiration pneumonia, significant choking, weight loss, or nutritional compromise were reported. Formal videofluoroscopic swallowing assessment was not performed. Although previously misdiagnosed with Myasthenia Gravis (MG), his clinical progression--characterized by stable rather than fluctuating symptoms--prompted further investigation. There was no family history of ptosis, ophthalmoplegia, muscle weakness, neuromuscular disease, recurrent falls, or unexplained motor disability among first- or second-degree relatives. Gross motor development was mildly delayed, with independent walking achieved at 15 months. Fine motor skills, language acquisition, social interaction, and academic performance were age-appropriate, and no cognitive or behavioral concerns were identified.

On examination, the patient is alert and cooperative, with normal mental status and age-appropriate language, attention, and behavior. Cranial nerve examination demonstrated severe symmetric bilateral ptosis. Extraocular movements were severely restricted in all directions, most prominently in upgaze and lateral gaze, with approximately 70-80% limitation of horizontal movements and 90% limitation of upward gaze bilaterally, consistent with external ophthalmoplegia. According to parental reports and serial clinical assessments, these ocular findings remained largely stable without significant progression over time. Pupils were equal, round, and reactive to light. Facial sensation was intact, and facial movements were symmetric. Hearing was grossly intact. Palatal elevation was symmetric, and the tongue was midline without atrophy or fasciculations. Muscle strength was assessed using the Medical Research Council (MRC) grading scale. Shoulder abduction and hip flexion were graded 4/5 bilaterally, while elbow flexion, wrist extension, knee extension, and distal muscle groups remained 5/5. The patient demonstrated difficulty rising from a seated position on the floor and utilized a compensatory manoeuvre consistent with a positive Gowers’ sign. Muscle tone was normal throughout, with no evidence of muscle atrophy, fasciculations, or abnormal involuntary movements. Deep tendon reflexes were normal and symmetric in the upper and lower extremities. Plantar responses were flexor bilaterally. Sensory examination was normal to light touch and proprioception. Coordination was intact. Gait was normal for age, without ataxia, a Trendelenburg pattern, or instability. Overall, the neurological examination was notable for severe external ophthalmoplegia with bilateral ptosis and a myopathic pattern of weakness characterized by mild upper extremity weakness, proximal lower extremity weakness, and a positive Gowers’ sign, with otherwise preserved reflexes, sensation, coordination, gait, and cognitive function.

Initial metabolic screening investigations were unremarkable. Serum creatine kinase (CK) was 82 U/L (reference range: 30-200 U/L), indicating no biochemical evidence of active muscle fiber necrosis. A brain MRI was obtained to exclude structural central nervous system abnormalities and mitochondrial disorders that may present with ophthalmoplegia. Muscle MRI was considered; however, it was not performed because genetic testing provided a definitive molecular diagnosis and the family declined additional investigations.

The weakness demonstrated slow progression over time, with increasing difficulty rising from the floor and climbing stairs, although the patient remained independently ambulatory. The combination of preserved sensation, normal reflexes, proximal-predominant weakness, and a positive Gowers' sign supported a primary myopathic process rather than a neuropathic disorder. A summary of the patient’s clinical, laboratory, imaging, and genetic findings is provided in Table 1.

**Table 1:** Summary of clinical, laboratory, imaging, and genetic findings. This table summarizes the key demographic, clinical, neurological, radiological, and genetic features of a six-year-old boy with congenital ptosis, external ophthalmoplegia, and proximal muscle weakness. Findings include a myopathic pattern of weakness with preserved reflexes and sensation, a normal brain MRI, mildly elevated-normal CK levels, and intermittent dysphagia without aspiration. Genetic analysis identified a homozygous MYH2 c.1124A>C (p.Gln375Pro) variant, with parental heterozygous carrier status and ACMG classification as a variant of uncertain significance (PM2 + PP1 + PP3). CK: creatine kinase; MRC: Medical Research Council.

Category	Findings
Demographics	six-year-old boy, consanguineous parents
Presenting symptoms	Congenital ptosis, external ophthalmoplegia, and proximal muscle weakness
Motor development	Mild delay (independent walking at 15 months)
Cranial nerves	Severe bilateral ophthalmoplegia (~70-90% limitation) and symmetric ptosis
Limb strength (MRC)	Proximal upper limbs: 4+/5; proximal lower limbs: 4/5; distal: 5/5
Reflexes	Normal and symmetric
Sensation	Intact
Coordination	Normal
Gait	Myopathic (positive Gowers’ sign)
CK level	82 U/L (normal range 30-200 U/L)
MRI brain	Normal
Swallowing	Mild intermittent dysphagia and no aspiration
Genetic finding	Homozygous MYH2 c.1124A>C (p.Gln375Pro)
Segregation	Parents heterozygous; sibling negative for homozygosity
ACMG classification	Variant of uncertain significance (PM2 + PP1 + PP3)

**Figure 1:**
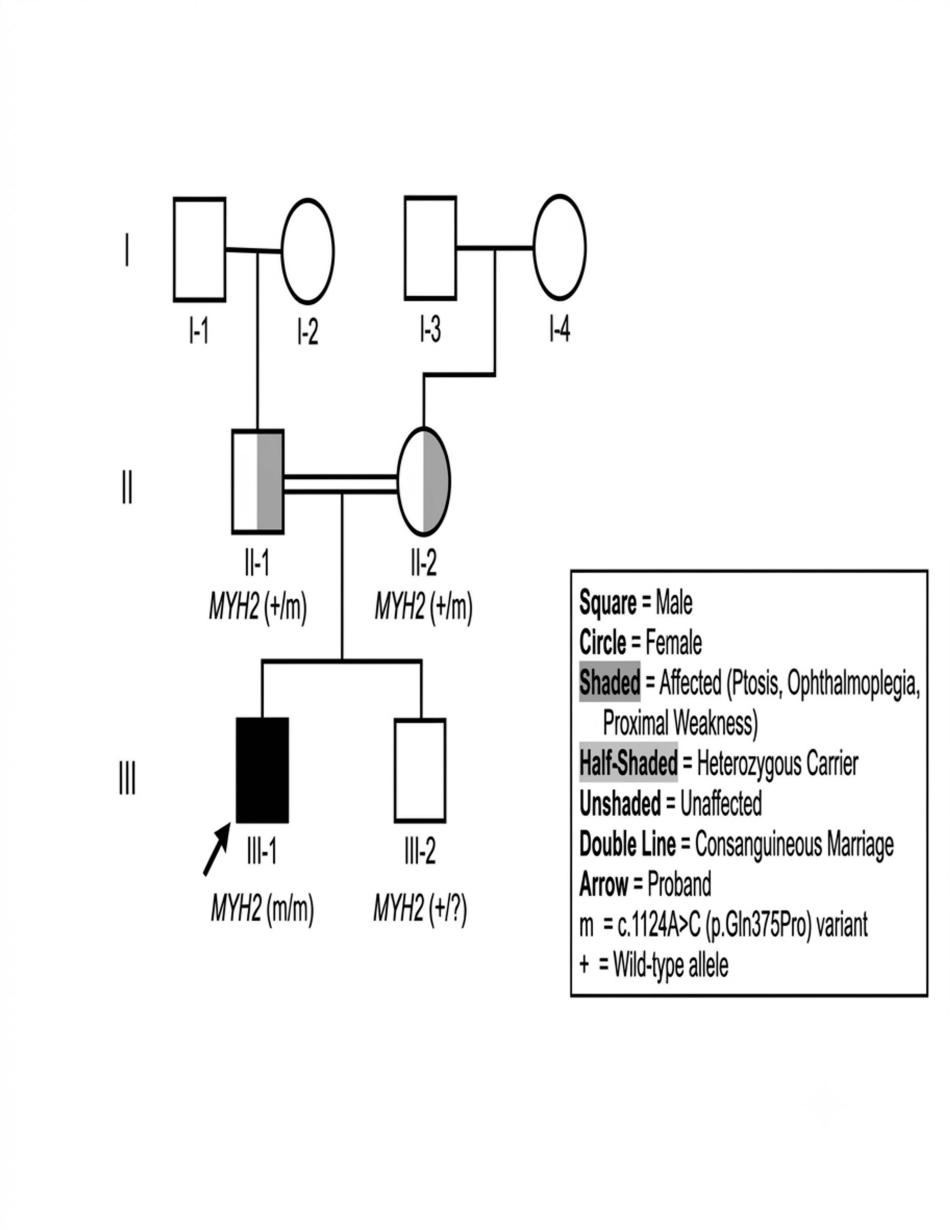
Pedigree analysis of the MYH2 variant. This pedigree demonstrates autosomal recessive inheritance in a consanguineous three-generation family. The parents (II-1 and II-2) are asymptomatic heterozygous carriers of the MYH2 c.1124A>C (p.Gln375Pro) variant, confirmed by Sanger sequencing. The proband (III-1) is homozygous for the variant and presents with congenital ptosis, severe external ophthalmoplegia, and proximal muscle weakness, consistent with recessive MYH2-related myopathy. The younger sibling (III-2) is clinically asymptomatic and has not undergone genetic testing; therefore, his genotype remains unknown (+/?). This segregation pattern supports autosomal recessive inheritance.

A muscle biopsy was not performed due to parental preference. Whole Exome Sequencing (WES) was performed and consistent with autosomal recessive proximal myopathy and ophthalmoplegia. Variant validation and family co-segregation analysis were performed using Sanger sequencing, which revealed that both parents are heterozygous for MYH2, p.Gln375Pro. The unaffected younger sibling, aged four, is clinically asymptomatic but has not undergone genetic testing. As shown in (Figure 1), the pedigree demonstrates autosomal recessive inheritance in a consanguineous family, with the proband (III-1) being the only affected individual and homozygous for the variant. Both parents are asymptomatic heterozygous carriers, while the sibling remains clinically unaffected. This segregation pattern supports recessive inheritance and strengthens the genotype-phenotype correlation.

The genomic DNA was quantified using Qubit v.3 (Life Technologies, Waltham, Massachusetts) and quality checked by gel electrophoresis. Library preparation was carried out using the TruSeq Capture Exome Kit (Illumina, San Diego, California). This kit provides coverage of 45Mb of exonic content. The probe set was designed to enrich 214,405 exons. After sequencing on NextSeq 500 (Illumina, San Diego, California), data were uploaded onto our server, and reads were aligned to the reference human genom (hg19) using the BWA aligner (Boston, Massachusetts). Prior to variant calling by GATK (Genome Analyisis Tool kit, Cambridge, Massachusetts), mapped reads (BAM format) went through preprocessing steps by removing PCR duplicates, realigning around indels, and recalibrating base quality. The final list of variants was annotated by ANNOVAR (Philadelphia, Pennsylvania) using several databases of minor allele frequency such as PopFreqMax, as well as variant effect predictors, such as SIFT, PolyPhen-2, and REVEL. Variants with low coverage, synonymous, predicted benign (SIFT, PolyPhen-2, and REVEL), MAF > 0.1% on gnomAD, PopFreqMax, and our Palestinian in-house database were filtered out.

Genetic analysis identified a homozygous variant of uncertain significance (VUS) in the MYH2 gene: c.1124A>C; p.(Gln375Pro). Segregation analysis confirmed that both parents are heterozygous carriers of the same variant, consistent with autosomal recessive inheritance. This specific gene is strongly associated with autosomal recessive proximal myopathy and ophthalmoplegia. No copy number variation (CNV) clinically related to the described phenotype was identified through NGS data analysis. Mean sequencing depth across target regions was 115×, with 98.4% of targeted bases achieving coverage greater than 20×. No other pathogenic or likely pathogenic variants relevant to the patient's phenotype were identified following variant filtering and multidisciplinary review.

Computational in silico analyses supported a potential deleterious effect of the MYH2 p.Gln375Pro variant. PolyPhen-2 predicted it to be probably damaging, SIFT indicated a deleterious substitution, and MutationTaster classified it as disease-causing, while REVEL scores were consistent with a pathogenic impact. The affected residue is highly conserved across vertebrate species, further underscoring its functional importance. In addition, the variant is absent from gnomAD v4.1, a local Palestinian in-house database, and other population datasets and shows familial co-segregation with disease (PM2, PP1, and PP3). Collectively, these data support a possible pathogenic role, although definitive confirmation requires functional validation.

According to ACMG/AMP guidelines, the c.1124A>C (p.Gln375Pro) variant meets the PM2 criterion due to its absence from population databases, PP1 based on familial co-segregation, and PP3 because multiple in silico prediction tools support a deleterious effect. Nevertheless, the available evidence is insufficient for classification as likely pathogenic, and the variant remains categorized as a VUS owing to the absence of functional validation, previously established pathogenic variants affecting the same residue, and histopathological confirmation.

## Discussion

Recessive MYH2-associated myopathy is a recognized form of congenital myopathy characterized by mild-to-moderate proximal muscle weakness, ptosis, and external ophthalmoplegia. The disorder typically presents in childhood with non-fluctuating weakness, a clinical feature that helps distinguish it from neuromuscular junction disorders such as MG. Histopathologically, recessive MYH2 myopathy demonstrates a characteristic pattern with marked reduction or near absence of type 2A muscle fibers, reflecting impaired expression of the MyHC-IIa isoform encoded by MYH2. Muscle biopsies frequently show relative predominance of type 1 fibers, consistent with selective loss of fast type 2A fibers [1].

Compared with dominant MYH2-associated disease, recessive cases generally present earlier in life, demonstrate more prominent ophthalmoplegia, and typically exhibit loss of type 2A fibers without rimmed vacuoles [1]. Dominant cases often present later and may show greater pathological heterogeneity [3].

The homozygous c.1124A>C; p.(Gln375Pro) variant identified in our patient is currently classified as a VUS. Interpretation of missense variants in MYH2 remains challenging because pathogenicity depends on multiple factors, including the biochemical properties of the substituted amino acid, its location within conserved functional domains of the MyHC-IIa protein, and its downstream effects on muscle fiber structure and composition. Previously reported pathogenic recessive MYH2 variants include truncating, splice-site, and missense mutations that ultimately result in loss or severe reduction of MyHC-IIa expression and depletion of type 2A fibers on muscle biopsy, correlating with the clinical phenotype of ophthalmoplegia and proximal muscle weakness [1].

Several case reports and case series have described MYH2-related myopathies presenting with the core features of external ophthalmoplegia, ptosis, and proximal muscle weakness, highlighting the phenotypic variability of this rare disorder. Early reports of recessive MYH2 myopathy documented patients with homozygous or compound heterozygous mutations presenting with mild-to-moderate weakness and complete absence of type 2A fibers on muscle biopsy, establishing ophthalmoplegia as a hallmark clinical feature of the recessive form [1]. Subsequent reports have identified additional recessive mutations associated with congenital myopathy, facial involvement, and ophthalmoplegia, supporting a loss-of-function disease mechanism [4]. Rare de novo and dominant missense mutations have also been described and may present with congenital muscle weakness, dysphagia, and external ophthalmoplegia, further broadening the genotypic and phenotypic spectrum of MYH2-associated disease [5]. More recent reports document both classical and atypical presentations, including bulbar involvement or adult-onset disease with variable severity, underscoring the importance of careful clinical and genetic correlation in patients harboring MYH2 variants [6].

Recent literature over the past decade has further refined the clinical and genetic spectrum of MYH2-related myopathy. Contemporary case series and systematic reviews have confirmed that the majority of patients present in childhood with external ophthalmoplegia, ptosis, and variable proximal muscle weakness, although adult-onset cases have also been increasingly recognized in smaller subsets of patients [7,8]. In addition, recent reports have expanded the phenotypic variability of both recessive and dominant forms, demonstrated overlapping clinical features, and emphasized that ophthalmoplegia may be present across the disease spectrum irrespective of inheritance pattern [9]. Advances in molecular diagnostics and next-generation sequencing have also contributed to improved recognition of atypical and milder presentations, supporting the concept that MYH2-related disorders represent a broader clinicogenetic continuum rather than strictly distinct entities [10].

The initial diagnosis of MG was based on ptosis and ophthalmoplegia. However, the absence of symptom fluctuation, lack of fatigability, normal deep tendon reflexes, persistently stable weakness, and negative acetylcholine receptor and MuSK antibodies argued against a neuromuscular junction disorder. Repetitive nerve stimulation showed no decremental response.

Our patient closely mirrors the classical recessive MYH2 phenotype reported in the literature, including congenital bilateral ptosis, severe ophthalmoplegia, preserved reflexes, normal cognition, and predominantly proximal muscle weakness. However, unlike many previously reported cases, histopathological confirmation through muscle biopsy was unavailable because muscle biopsy was declined by the family due to the financial constraints, representing an important limitation of the present report, which could have demonstrated the characteristic reduction or absence of type 2A fibers and loss of MyHC-IIa expression described in MYH2-associated myopathy.

The p.Gln375Pro variant differs from previously reported MYH2 mutations by affecting a highly conserved residue within the motor domain of MyHC-IIa, a region critical for ATP-dependent force generation. To our knowledge, this specific amino acid substitution has not previously been reported in the literature or population databases.

In the present case, segregation of the c.1124A>C variant with disease in the affected child, combined with its absence in a clinically unaffected sibling and heterozygous carrier status in both consanguineous parents, supports an autosomal recessive inheritance pattern and strengthens the likelihood of pathogenicity. Segregation analysis represents an important supportive criterion in variant interpretation. Nevertheless, additional data would further clarify the pathogenic role of this variant. A muscle biopsy demonstrating reduction or absence of type 2A fibers with loss of MyHC-IIa expression would provide strong pathological correlation. Similarly, muscle MRI or functional studies examining transcript or protein expression could offer additional mechanistic insight. Long-term clinical follow-up of the patient and monitoring of at-risk family members may also help clarify disease progression and penetrance.

## Conclusions

Autosomal recessive MYH2-associated myopathy remains exceptionally rare, with only a limited number of genetically confirmed families reported worldwide. We describe a child with congenital bilateral ptosis, external ophthalmoplegia, and proximal muscle weakness in whom a previously unreported homozygous MYH2 missense variant was identified. Although currently classified as a VUS, the segregation pattern within the family, the clinical phenotype consistent with recessive MYH2 myopathy, and the variant’s location within a conserved region of the myosin motor domain support a possible disease association, although definitive pathogenicity remains to be established through functional studies and additional reported cases. Future functional analyses, together with the accumulation of further independent cases carrying the same or similar variants, will be essential to clarify the pathogenic role of this variant and strengthen genotype-phenotype correlations. This case further expands the genetic and clinical spectrum of MYH2-associated myopathy and highlights the importance of detailed clinical and molecular characterization in improving the interpretation of novel variants.
